# A decade of antimicrobial resistance research in social science fields: a scientometric review

**DOI:** 10.1186/s13756-020-00834-2

**Published:** 2020-11-04

**Authors:** Jiahui Lu, Anita Sheldenkar, May Oo Lwin

**Affiliations:** 1 School of New Media and Communication, Tianjin University, Tianjin, China; 2 Wee Kim Wee School of Communication and Information, Nanyang Technological University, Singapore, Singapore

**Keywords:** Antimicrobial resistance, Antibiotic resistance, Social science, One health, Bibliometrics, Interdisciplinary

## Abstract

**Background:**

Though social sciences are expectedly instrumental in combating antimicrobial resistance (AMR), their research on AMR has been historically lacking.

**Objectives:**

This study aims to understand the current academic literature on AMR within the social science field by investigating international contributions, emerging topics, influential articles, and prominent outlets, to identify research gaps and future directions.

**Methods:**

Bibliometric data of 787 peer-reviewed journal articles published in the period of 2010 to 2019 were extracted from the Social Science Citation Index in the Web of Science database. Bibliographic networks of the extracted articles were examined.

**Results:**

Social science research on AMR has grown rapidly in the past 5 years. While western developed countries contributed the most to the field in the past decade, research within developing regions such as Asia and Africa have increased in the last 2 years. Social sciences have been contributing to AMR research in several different domains from surveillance and risk assessment of AMR, to promotions of appropriate use of antimicrobials in primary care and clinical settings. Though the idea of one health has been incorporated into research on AMR within the medical and microbial science fields, it has not been well recognized by social sciences.

**Conclusion:**

Social science research on AMR is a new, while rapidly developing, research area that requires continued and intense global efforts from an interdisciplinary and one health approach. Research on social issues surrounding AMR transmissions between human, animal, and environments should be emphasized in the future.

## Introduction

Antimicrobial Resistance (AMR) occurs when microorganisms develop resistance to antimicrobial medications such as antivirals and antibiotics. AMR is increasingly becoming a global threat, both economically as well as in terms of health. AMR can lead to the treatments becoming ineffective, making it more difficult to treat infections, and can then cause an increase in the risk of the disease spreading [1]. Particularly, diseases such as tuberculosis, malaria, HIV and Influenza are developing more drug resistant cases, leading to higher healthcare costs and a longer duration of illness. By 2050, the impact of AMR is estimated to lead to 10 million deaths per year, with a global economic cost of $100 trillion [2]. Within the European Union alone, the average yearly additional costs caused by AMR between 2015 and 2030 is estimated to be $252,215 USD per 100,000 persons per year [3].

Several factors have been found to cause AMR. Human use of antimicrobial drugs, particularly antibiotics, is recognized as one of the primary drivers of AMR. Antibiotics are among the most familiar of medicines by the public, and the global consumption of antibiotics has been estimated at more than 35 billion daily doses at 2015 [4]. Several studies have found a positive association between antimicrobial use and AMR [5–7]. Although in the past AMR research has focused on direct public consumption of antimicrobial drugs, drugs use in livestock are an increasingly concerning issue. Antibiotics are an integral part of industrial agriculture to ensure healthy livestock and promotion of growth [8]. The Food and Agriculture Organization (FAO) expects that two-third of the future growth of antimicrobial use will be linked to animal production [9]. Human consumption of these animals can lead to resistant microorganism transmission between hosts. This can then in turn impact the wider environment through animal and human waste affecting soil and land.

Different approaches have been proposed to tackle AMR. One effective way is to provide interventions through antimicrobial stewardship programmes, which aims to promote the appropriate use of antimicrobials [10, 11]. On a larger scale, the idea of ‘one health’ has been increasing in popularity for combating AMR. One health is an approach for the improvement of public health through the promotion and collaboration of multidisciplinary areas [12]. Within the AMR space, this typically involves sectors from animal health, human health, food safety, and environment working together to prevent the spread of resistant microorganisms. The World Health Organisation (WHO) highlighted the one health approach as a guide for the global action plan and framework on AMR [13, 14].

Social sciences, encompassing a wide range of academic disciplines, such as sociology, psychology, communication, and economics, are expected to play a significant role in antimicrobial stewardship and the one health approach. For example, antimicrobial stewardship often requires theories, frameworks, and methods from behavioural and psychological sciences at a micro level. The one health approach also inspires social sciences to address AMR from the societal, historical, and economic perspectives. However, within the social science field, academic literature on AMR has historically been lacking [15]. It is only in recent years that the values of social sciences are increasingly recognised in AMR research [16–18].

As the global efforts to reduce AMR rapidly increases, and social science is becoming more prominent in the field, existing gaps and future directions need to be identified. Therefore, this study investigates the social science literature surrounding this topic and how the landscape has changed and developed over the last decade. We aim to analyse the bibliometric data of the literature in the field via network analyses. Frid-Nielson et al. [15] have utilized similar techniques and revealed that the social science contribution to AMR is weak and peripheral compared to its contributions to other research areas such as climate change. However, our study bears significant differences and extensions from their study. First, our study focuses on literature within the last decade instead of looking at a wider scope of several decades. This is to ensure that the most recent development of social science AMR research are highlighted by avoiding the impact of early clinical and medical literature on network analyses. Second, we will perform more in-depth analyses than the previous study, including the presentation of co-authorship networks and keyword co-occurrence networks. We will also perform analyses based on the influential articles within the field in the last decade. These analyses are critical as they can reveal the most recent state of international contributions and emerging research topics in the field, allowing further identification of research gaps and future directions.

## Methods

We followed the PRISMA guidelines [19] for conducting the review and reporting results. The Social Science Citation Index (SSCI) in the Web of Science (WoS) database was selected as the data source for our review. SSCI is a reputable scientific index that covers over 3000 social sciences journals across more than 50 disciplines. In addition, the hosting WoS database provides structured and consistent indexes of authorships and references and is well respected within academia. The index allows wide access of social science research on AMR and relatively accurate network analysis of authorships and co-citations.

A topic search in SSCI for “antimicrobial resistan*” and “antibiotic resistan*” published from 2010 to 2019 was conducted on Jan 14, 2020. Inclusion criteria included publications in English and limits were set to full-text peer-review journals to ensure the accuracy of follow-up co-citation analyses. The search produced 787 unique articles published in 294 journals with no duplicates. Bibliographic data including full reference lists of each article were extracted. Data of all articles were included in the analysis.

VOSviewer is a software tool that was used to analyze and visualize the bibliographic networks. VOSviewer can provide distance-based bibliometric maps in which the strength of the relation between two items can be visualized as the distance between the two items, allowing easy understanding and visualization of clustering [20]. Co-authorship analysis based on countries were conducted to showcase the current state of international contributions. The keyword co-occurrence network was examined to explore the research landscape and understand research trajectories. Top cited and co-cited articles were briefly reviewed to detail the existing landscape, complementing the keyword network analysis.

Finally, the citation and co-citation network of journals were examined. The citation network, where a connection is demonstrated when a journal was cited by another journal, is used to reveal the knowledge flow in AMR social science research. The co-citation network, where a connection is demonstrated when a journal was co-cited with another journal by a research document, often indicates the topic similarity between journals. In this study, we use the co-citation network to reveal the knowledge foundation of the research field, as it can present and cluster all journals that were cited by research documents in the current dataset.

## Results

### Numbers of publications

**Fig. 1:**
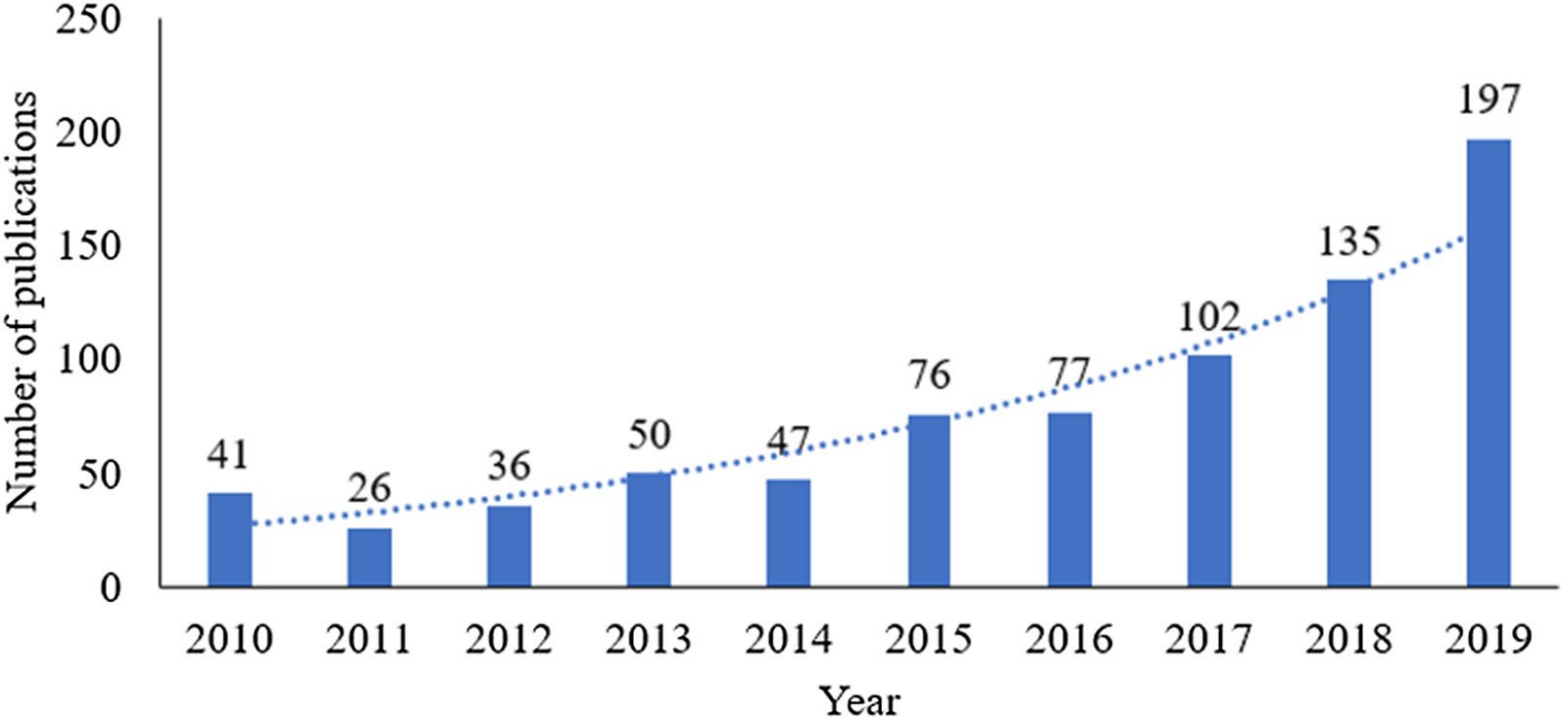
Number of publications on AMR social science research in the last decade

As demonstrated in Fig. 1, the number of publications on AMR social science research grew rapidly since 2016. The AMR publications tripled from 77 in 2016 to 197 in 2019. A quadric function fitted the growing patter well and thus it is expected that the AMR social science research will continue to grow in this new decade.

### International contributions

Table 1 shows the top 15 countries that contributed to the field. The United States of America (USA) participated in more than one-third of the articles, followed by England. We categorized countries based on their income-level indicated by the World Bank [21]. High-income countries constituted 11 of the top 15 contributing countries. A closer analysis showed that 79.2% (N = 623) of corresponding authors within the extracted articles were from high income countries, while the figure was 14.9% (N = 117) for upper-middle income countries, 4.32% (N = 34) for lower-middle income countries, and 1.65% (N = 13) for low income countries. In addition, the latter three income groups were involved in only 31.2% of all articles. The findings indicate that high income countries were leading the AMR social science research, and there is a lack of research in less developed countries.

**Table 1:** The top 15 countries among the 112 countries contributed to the field

Country	Documents	Citations	Income level	Continent	WHO region
USA	263	4639	High	North America	Region of the Americas
England	159	3760	High	Europe	European Region
Australia	64	792	High	Australia	Western Pacific Region
Sweden	52	2071	High	Europe	European Region
Canada	42	1911	High	North America	Region of the Americas
Netherlands	42	638	High	Europe	European Region
China, mainland	40	410	Upper middle	Asia	Western Pacific Region
Germany	33	542	High	Europe	European Region
Switzerland	29	2554	High	Europe	European Region
India	28	1891	Lower middle	Asia	South-East Asia Region
Italy	26	170	High	Europe	European Region
South Africa	26	1736	Upper middle	Africa	African Region
Scotland	24	1671	High	Europe	European Region
Belgium	21	1777	High	Europe	European Region
Thailand	21	1435	Upper middle	Asia	South-East Asia Region

A total of 112 countries collaborated on the 787 AMR articles published in SSCI. Particularly, forty-three of the countries co-authored more than 5 articles and 31 co-authored more than 10 articles. Approximately one-third of the articles (N = 245) were co-authored by different countries, with only 3% of articles co-authored by more than five countries. The above findings reflect AMR as a global issue because a large number of countries have been involved in the academic efforts. However, the findings also indicate that there were not as many international collaborations as expected to be in AMR social science research.

**Fig. 2:**
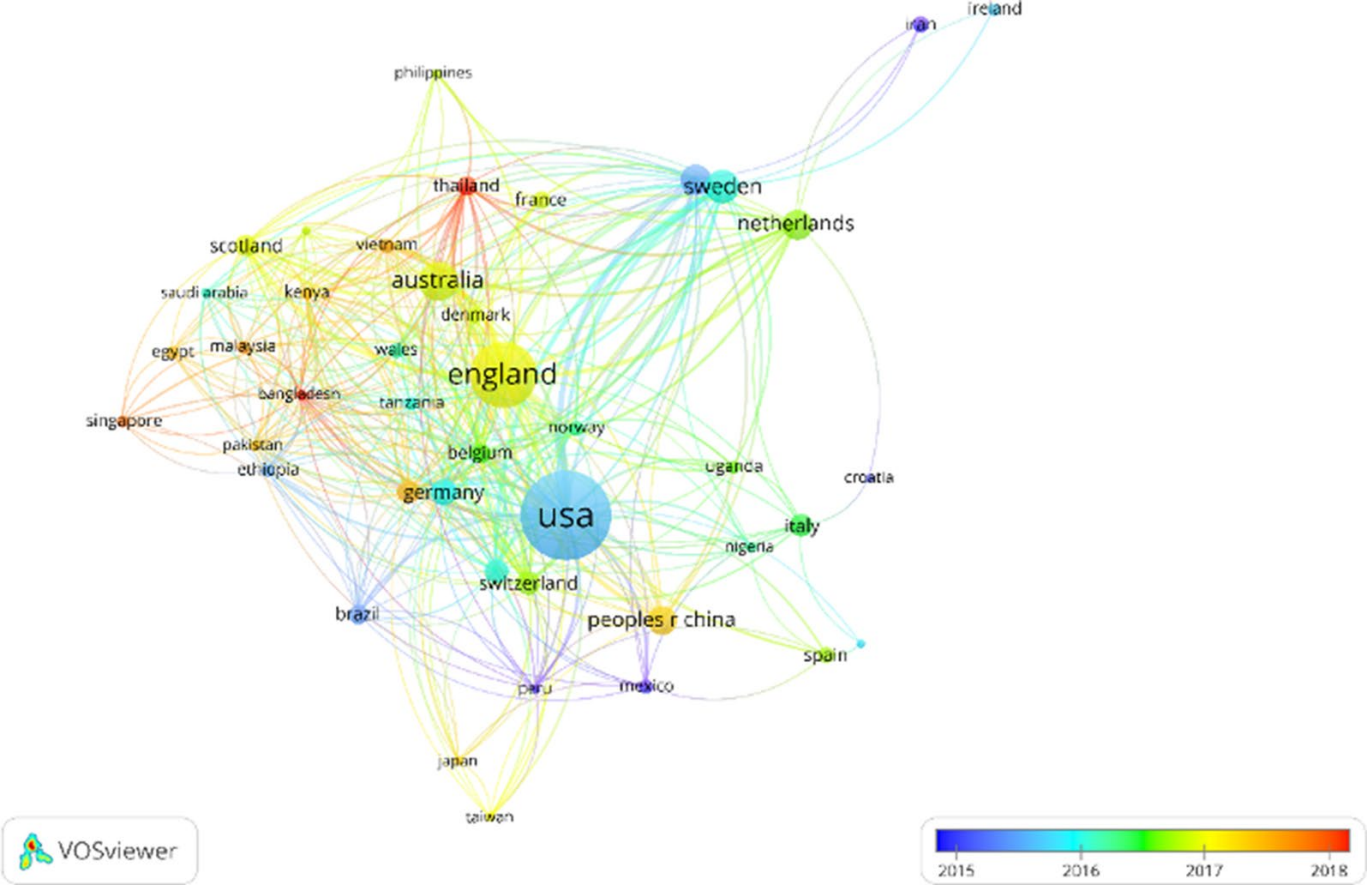
Co-authorship network of countries with more than five documents. The node size indicated the size of publications, while the node colour was scaled to the averaged publication year. Edges between nodes indicated co-authorships between countries. The thicker an edge is, the more the countries co-authored

Figure 2 shows the co-authorship network between countries with more than 5 articles. Each node indicates a country. The node size indicates the size of publications, while the node colour is scaled to the averaged publication years. Edges between nodes indicates the co-authorships between countries. It shows that USA and England are located at the centre of the graph, suggesting that they were the two countries that collaborated the most with other countries and led the AMR social science research. Countries from Asian, Africa, and South America located at the periphery of the graph with smaller nodes, indicates research from these countries are lacking in the AMR social science discourses.

Surprisingly, countries from the same WHO regions [22] were not always clustered together and often far from each other. For example, in countries from the Western Pacific Region, though Singapore and Malaysia were clustered, their collaborations were closer with Egypt, Bangladesh, Pakistan, and Ethiopia than China and Japan. Likewise, Philippines collaborated more with Thailand and Vietnam than other countries in the same region. Similar patterns were also observed for other WHO regions such as the European Region and the Eastern Mediterranean Region. This suggests that regional actions, coordination, and collaborations under WHO are not intense at the academic level.

In addition, the colour distribution of the nodes shows that articles within countries from Asia (e.g., Thailand, Singapore, Bangladesh) and Africa (e.g., Egypt, Kenya) have an average publication year of 2017–2018. This indicates that research coming from Asia and Southern Africa in the social science field has increased in the past two years. Notably, the research from Southern America seems to be relatively small and stagnant, with the majority of articles being published around 2015.

### Emerging topics

**Fig. 3:**
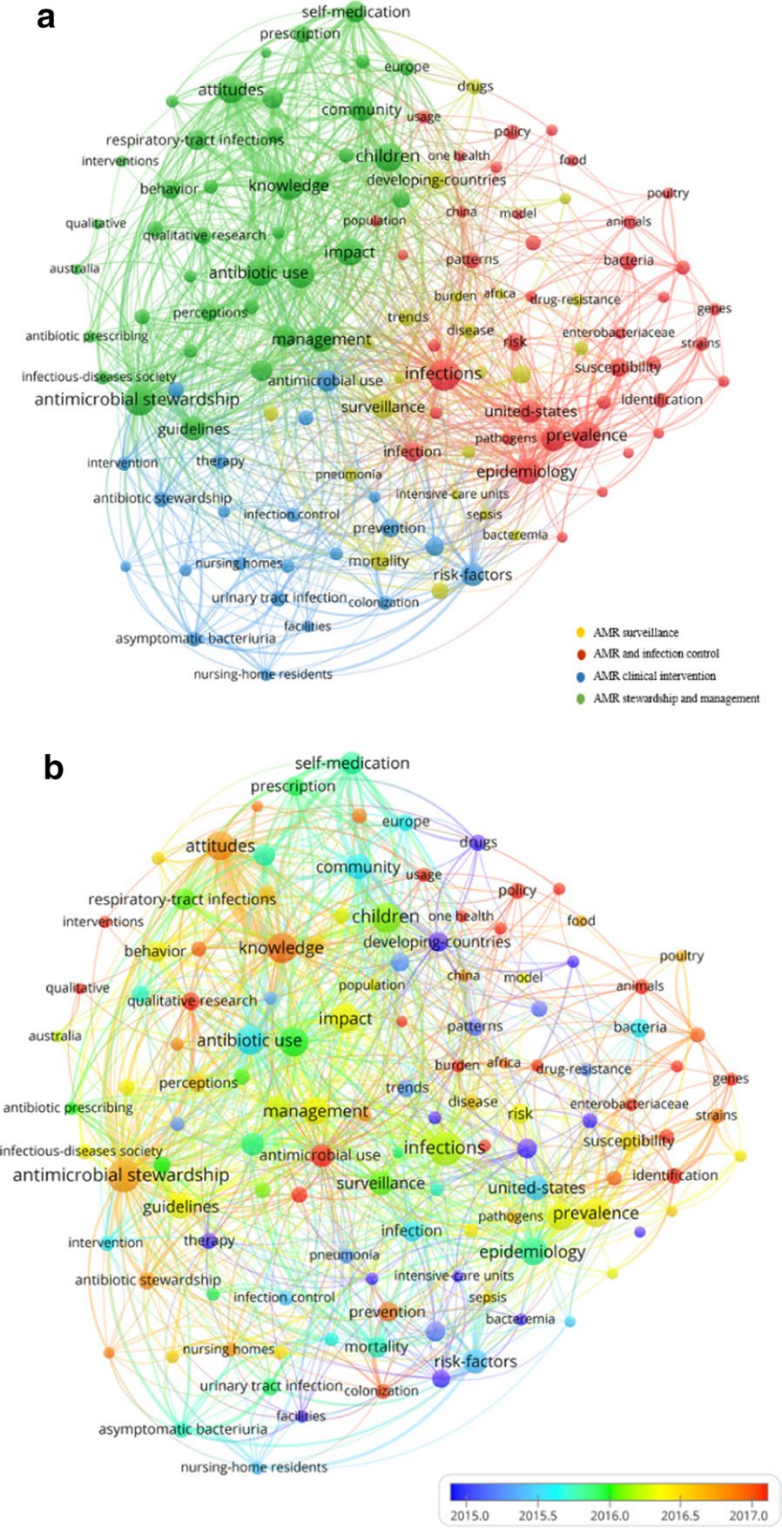
Co-occurrence network of keywords. The node size indicated the number of occurrences of a keyword. Edges between nodes indicated co-occurrences between keywords. The thicker an edge is, the more a keyword co-occurred with the other keyword. The node colour of **a** indicates the category of a node. The node colour of **b** was scaled to the averaged publication year

Co-occurrence of article keywords was analysed and visualised to showcase the topical clustering and their development trajectories (Fig. 3). In Fig. 3, the node size indicated the number of occurrences of a keyword. Edges between nodes indicated co-occurrences between keywords. The thicker an edge is, the more a keyword co-occurred with the other keyword. The node colour of Fig. 3a indicates the category of a node. The node colour of the Fig. 3b was scaled to the averaged publication year. Table 2 lists the most prevalent keywords for each cluster based on Fig. 3a.

**Table 2:** Top keywords for research clusters

Red cluster	Freq	Yellow cluster	Freq	Blue cluster	Freq	Green cluster	Freq
Infections	69	Surveillance	39	Risk-factors	39	Antimicrobial stewardship	67
Prevalence	52	Mortality	28	Antimicrobial use	33	Children	57
Epidemiology	46	*Staphylococcus aureus*	27	Resistance *Staphylococcus aureus*	27	Knowledge	56
*Escherichia coli*	45	Developing-countries	25	Prevention	25	Antibiotic use	53
United States	39	Blood-stream infections	22	Program	20	Attitudes	52
Infection	28	Trends	22	Antibiotic stewardship	19	Care	52
Risk	26	Outcomes	18	Urinary tract infection	19	Impact	52
Susceptibility	25	Disease	17	Asymptomatic bacteriuria	17	Management	47
Bacteria	23	Drugs	17	Stewardship	17	Community	41
Patterns	19	Emergence	15	Therapy	17	Guidelines	41

As demonstrated in Fig. 3a, three major clusters were formed, with another cluster merged and overlapped with the others. The clusters presented in red and yellow represented the research on AMR surveillance, infection control, and risk assessment from a medical perspective. The cluster in blue presented the applied clinical research for intervention and stewardship programs about AMR. Finally, the green cluster indicated the social and behavioural science research on antimicrobial stewardship and management, and on knowledge, attitudes, and behaviours regarding antimicrobial prescription and use.

Interestingly, keywords indicating environmental research and one health (e.g., animals, genes, food) were predominantly clustered within AMR epidemiology and surveillance research (Red cluster), indicating that they were more connected with AMR clinical surveillance than other research topics. They were also located peripherally at the connection area between social science research (Green cluster) and the clinical surveillance research. This suggests that the environmental and one health research has the potential to integrate the two research areas. However, keywords of one health research in the current dataset were relatively isolated as they demonstrated less dense connections with other keywords or clusters. This implies that the one health approach is at the periphery of the AMR social science discourse.

Figure 3b reveals that over time, research topics have developed from AMR surveillance and risk assessment, applied clinical AMR management (indicated by blue nodes), to social and behavioural science research on antimicrobial stewardship (indicated by green nodes), and to the most recently, the one health approach including management of AMR in animal and environmental sectors (indicated by red nodes).

### Influential articles

To further understand the literature surrounding topics emerging from the keyword co-occurrence analysis, citation and co-citation analyses of references were conducted to identify influential articles in each topic. Tables 3 and 4 lists the 20 most cited and co-cited articles, respectively. The top cited articles reflect the current research interests while the top co-cited articles highlight the foundations of the field that can be cited by different areas of research.

**Table 3:** Top 20 cited articles in AMR social science research

Documents	Citations	Journal, Year	Title
Laxminarayan et al. [23]	1318	Lancet Infect Dis	Antibiotic resistance-the need for global solutions
Allegranzi et al. [24]	696	Lancet	Burden of endemic health-care-associated infection in developing countries: systematic review and meta-analysis
Hirsch and Tam [25]	174	Expert Rev Pharm Out	Impact of multidrug-resistant Pseudomonas aeruginosa infection on patient outcomes
Charani et al. [26]	166	Clin Infect Dis	Understanding the Determinants of Antimicrobial Prescribing Within Hospitals: The Role of “Prescribing Etiquette”
Dheda et al. [27]	163	Lancet Respir Med	The epidemiology, pathogenesis, transmission, diagnosis, and management of multidrug-resistant, extensively drug-resistant, and incurable tuberculosis
Unemo et al. [28]	144	Lancet Infect Dis	Sexually transmitted infections: challenges ahead
Maron et al. [29]	133	Glob Health	Restrictions on antimicrobial use in food animal production: an international regulatory and economic survey
Pruden [30]	114	Environ Sci Technol	Balancing Water Sustainability and Public Health Goals in the Face of Growing Concerns about Antibiotic Resistance
Roberts et al. [31]	93	Med Care	Costs Attributable to Healthcare-Acquired Infection in Hospitalized Adults and a Comparison of Economic Methods
Marks et al. [32]	76	Lancet Glob Health	Incidence of invasive salmonella disease in sub-Saharan Africa: a multicentre population-based surveillance study
Ewig et al. [33]	75	Thorax	Nursing-home-acquired pneumonia in Germany: an 8-year prospective multicentre study
Togoobaatar et al. [34]	74	Bull World Health Organ	Survey of non-prescribed use of antibiotics for children in an urban community in Mongolia
Broom et al. [35]	71	Soc Sci Med	Cultures of resistance? A Bourdieusian analysis of doctors' antibiotic prescribing
Adriaenssens et al. [36]	69	BMJ Qual Saf	European Surveillance of Antimicrobial Consumption (ESAC): disease-specific quality indicators for outpatient antibiotic prescribing
Wirtz et al. [37]	63	Rev Panam Salud Publica	Trends in antibiotic utilization in eight Latin American countries, 1997–2007
Ardal et al. [38]	61	Lancet	International cooperation to improve access to and sustain effectiveness of antimicrobials
Lucas et al. [39]	59	Scand J Prim Health Care	A systematic review of parent and clinician views and perceptions that influence prescribing decisions in relation to acute childhood infections in primary care
Agarwal and Sankar [40]	59	Lancet Glob Health	Characterisation and antimicrobial resistance of sepsis pathogens in neonates born in tertiary care centres in Delhi, India: a cohort study
Luepke et al. [41]	57	Pharmacotherapy	Past, Present, and Future of Antibacterial Economics: Increasing Bacterial Resistance, Limited Antibiotic Pipeline, and Societal Implications
Phillips et al. [42]	55	BMC Geriatr	Asymptomatic bacteriuria, antibiotic use, and suspected urinary tract infections in four nursing homes

**Table 4:** Top 20 co-cited articles in AMR social science research

Documents	Co-citation	Journal	Title
WHO [13]	71	WHO	Global action plan on antimicrobial resistance
WHO [43]	56	WHO	Antimicrobial resistance: global report on surveillance
Laxminarayan et al. [23]	53	Lancet Infect Dis	Antibiotic resistance-the need for global solutions
Goossens et al. [44]	51	Lancet	Outpatient antibiotic use in Europe and association with resistance: a cross-national database study
Dellit et al. [45]	38	Clin Infect Dis	Infectious Diseases Society of America and the Society for Healthcare Epidemiology of America Guidelines for Developing an Institutional Program to Enhance Antimicrobial Stewardship
Costelloe et al. [5]	38	BMJ	Effect of antibiotic prescribing in primary care on antimicrobial resistance in individual patients: systematic review and meta-analysis
Broom et al. [35]	31	Soc Sci Med	Cultures of resistance? A Bourdieusian analysis of doctors' antibiotic prescribing
Morgan et al. [46]	29	Lancet Infect Dis	Non-prescription antimicrobial use worldwide: a systematic review
Okeke et al. [47]	29	Lancet Infect Dis	Antimicrobial resistance in developing countries. Part I: recent trends and current status
Huttner et al. [48]	28	Lancet Infect Dis	Characteristics and outcomes of public campaigns aimed at improving the use of antibiotics in outpatients in high-income countries
O'Neill [2]	28		Tackling drug-resistant infections globally: Final report and recommendations
Charani et al. [26]	26	Clin Infect Dis	Understanding the Determinants of Antimicrobial Prescribing Within Hospitals: The Role of "Prescribing Etiquette"
Radyowijati and Haak [49]	25	Soc Sci Med	Improving antibiotic use in low-income countries: an overview of evidence on determinants
van Boeckel et al. [50]	25	Lancet Infect Dis	Global antibiotic consumption 2000 to 2010: an analysis of national pharmaceutical sales data
Davey et al. [51]	23	Cochrane Database Syst Rev	Interventions to improve antibiotic prescribing practices for hospital inpatients
Levy and Marshall [52]	23	Nat Med	Antibacterial resistance worldwide: causes, challenges and responses
Loeb et al. [53]	22	Infect Control Hosp Epidemiol	Development of minimum criteria for the initiation of antibiotics in residents of long-term–care facilities: results of a consensus conference
van Boeckel et al. [54]	22	PNAS	Global trends in antimicrobial use in food animals
Butler et al. [55]	21	BMJ	Understanding the culture of prescribing: qualitative study of general practitioners' and patients' perceptions of antibiotics for sore throats
Holmes et al. [56]	21	Lancet	Understanding the mechanisms and drivers of antimicrobial resistance

#### AMR surveillance, diagnosis, mechanisms, and risk assessment

The knowledge and evidence gained from AMR surveillance and risk analytic research highlights the urgent need for social science research, which was found in half of the highly cited and co-cited articles. A global report by WHO in 2014 found very high rates of antimicrobial resistance for bacteria that cause common infections in healthcare and community settings around the world. [43] Specifically, it was found that common pathogens in developing countries have developed resistance at “unacceptable” levels, such as sepsis pathogens in India [40, 47].

Research has blamed increased and inappropriate use of antimicrobials for the resistance [5, 44, 52, 56]. Particularly, the global estimation of antibiotic consumption increased by 36% from 2000 to 2010 [50]. Surveillance research in clinical settings revealed a high prevalence of antimicrobial prescriptions around the world, many of which were prescribed erroneously [36, 57]. Non-prescription antimicrobial use is also common in most regions of the world, accounting for 19–100% of antimicrobial use [46]. It has been found that resistance leads to higher prevalence of drug-resistance infection, which is associated with worse clinical outcomes including higher mortality and morbidity and longer hospitalization [24, 25, 31, 33, 42]. Therefore, proper management of antimicrobial use to tackle drug-resistance infection has been called for.

#### AMR management and antimicrobial stewardship

Despite limited national and regional efforts in containing AMR in the early 2000s, [45, 48, 51, 53] after AMR was recognized as a global health crisis, collaborative international efforts have been called for and several frameworks have been proposed. For example, Ardal et al. [38] identified global policy gaps in tackling AMR in five areas, including surveillance, infection control, universal access, responsible use of antimicrobials, and research and development (R&D) for new drugs. Supported by the UK government, O’Neill (2016) proposed several interventions from an economic perspective such as public campaigns, promotion of vaccines and alternatives, and improvement of payment and recognition of healthcare workers [2]. Additionally, the WHO in 2017 proposed a global framework for combating AMR in three main areas including R&D, access, and stewardship [14].

Notably, recent calls of AMR management and stewardship have recognized the importance of R&D and appropriate use beyond humans. Particularly, in 2017, the WHO defined antimicrobial stewardship as an overarching term that includes promotion of appropriate use in human, animal, and plant health, from individual to global levels [14]. The scope of antimicrobial stewardship has been greatly broadened since then.

#### Determinants of human antimicrobial use

One of the keys to promoting appropriate antimicrobials use in human is to understand the determinants of antimicrobial prescription, dispensing, and community use. Research has revealed an antimicrobial prescribing culture in which immediate benefits (e.g., good doctor-patient relationship, avoidance of clinical risks) [35, 39, 49, 55], and pressures (e.g., peer norms and pressure) [26, 35], outweigh perceived long-term community risks of AMR across different countries at varied economic levels.

There is a relative lack of recent influential studies regarding antimicrobial dispensing and community use. However, some studies have found that lack of government regulation, economic incentives, and pressure to meet customers’ needs were major determinants of dispensing antimicrobials [49, 58]. Recent studies and reviews further showed that patient-related factors such as self-medication and the low socio-economic level of patients would increase dispensing without prescription [59, 60]. With regard to community use, evidence showed that lack of knowledge, lack of access to health facilities, and the tendency to self-medicate were associated with inappropriate antimicrobial use in less developed regions [49, 61]. This suggests that, cultural and societal factors may also be essential for understanding antimicrobial dispensing and community use.

#### Antimicrobial use beyond human, environmental research and the one health approach

In 2013, Laxminarayan and the colleagues [23] published a milestone article in the *Lancet Infectious Disease*, calling for global solutions to antibiotic resistance. The article summarized that resistance has spread worldwide and has highly burdened the world society with increasing health and economic costs. In addition, antibiotic use beyond humans is increasing and “a holistic, ecological, one health approach is needed (p. 9)”. Similarly, two years later, the WHO endorsed a global action plan on antimicrobial resistance, aiming at increasing awareness, strengthening knowledge, reducing infection, optimizing use of antimicrobials, and developing sustainable investment in new medicines through a one health approach [13].

However, social scientists have paid little attention to antimicrobial use beyond humans in the past decade, regardless of the global calls for the one health approach. Of the listed influential articles, only three articles are related to food animal and environmental science: two articles are targeted towards antimicrobial use in animal food products [29, 54] and one examines AMR in water environments [30]. The three articles suggest that residual antibiotics from agriculture and environments such as wastewater can lead to the development of resistant genes within pathogens which could then spread from animals and environments to humans. The limited attention given to AMR social science articles in animal and environmental sectors echoes findings of our keyword co-occurrence analysis that the one health approach is at the periphery of the AMR social science discourse.

### Prominent outlets

As we have identified four major topics in the literature of AMR social science research, we aim to further understand what outlets have been involved in this discussion. Table 5 demonstrates journals that published more than 1% of all articles on AMR social science research. Public health journals contributed the most to the publications, followed by medical journals that do not aim to publish social science research. A closer analysis on the full journal list demonstrated a notable absence of AMR research in general social science outlets in the fields of anthropology, education, economics, geography, sociology, psychology, and communication studies. Remarkably, research in outlets within these social science disciplines constituted only 6.2% of total publications.

**Table 5:** Journals publishing more than 1% of the articles on AMR social science research

	Frequency	Percent
International Journal of Environmental Research and Public Health	52	6.6
Plos One	22	2.8
Frontiers in Public Health	20	2.5
BMJ Global Health	14	1.8
Antimicrobial Resistance and Infection Control	12	1.5
Sexual Health	12	1.5
Iranian Journal of Public Health	12	1.5
BMC Public Health	11	1.4
BMJ Open	11	1.4
Preventive Veterinary Medicine	10	1.3
American Journal of Infection Control	10	1.3
Lancet Global Health	9	1.1
Infection Control and Hospital Epidemiology	9	1.1
Journal of Antimicrobial Chemotherapy	9	1.1
Public Health	9	1.1
Journal of Hospital Infection	9	1.1
Clinical Infectious Diseases	8	1
Social Science & Medicine	8	1
Journal of the American Geriatrics Society	8	1
Advances in Skin & Wound Care	8	1
Health Security	8	1

**Fig. 4:**
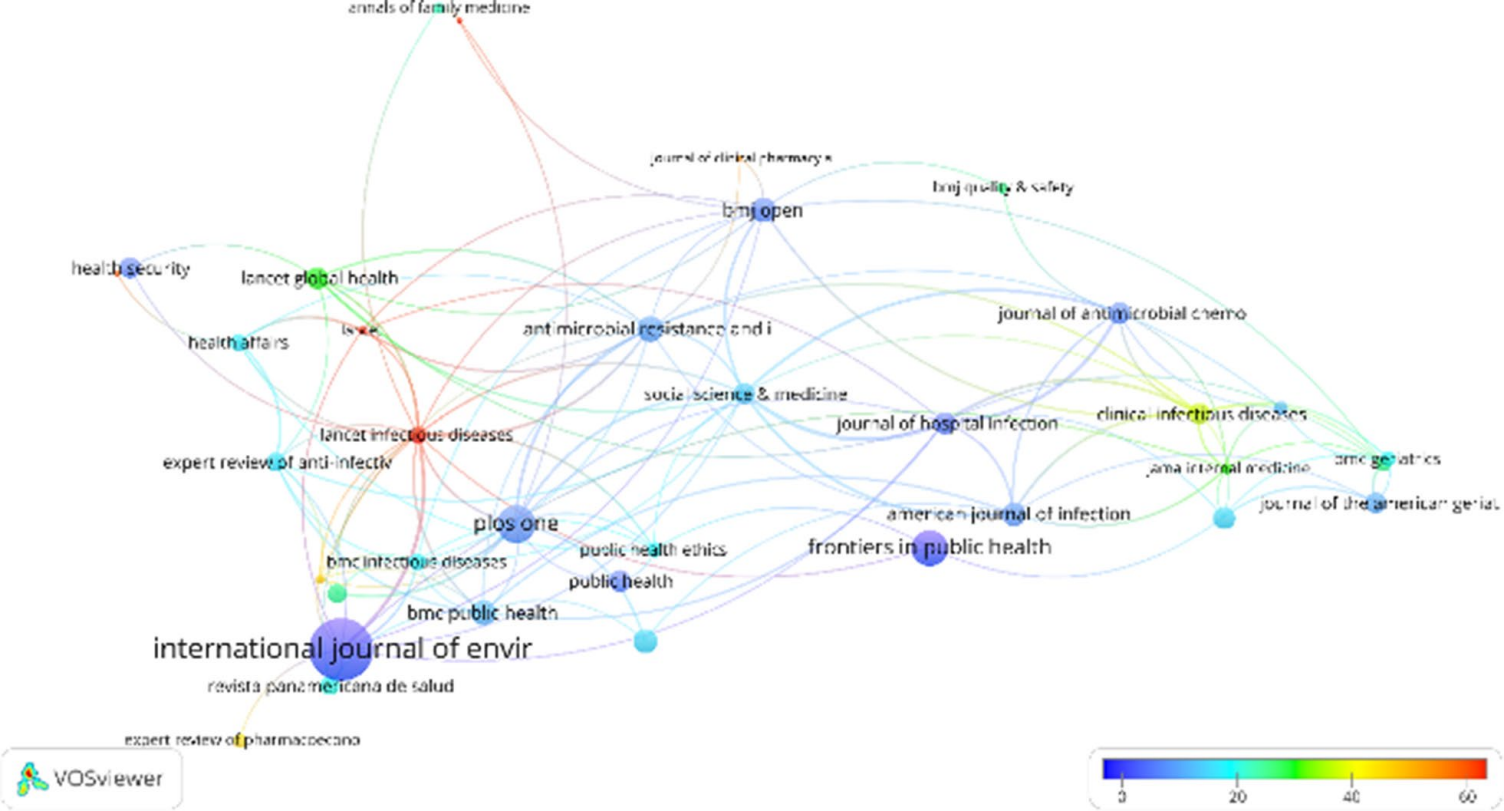
The citation network of journals that received more than 50 citations. The node size indicated the size of publications in a journal, while the node colour was scaled to the averaged citation per document. Edges between nodes indicated citation linkage between journals. The thicker an edge is, the more a journal cited or was cited the other journal

The citation network between 44 journals that received more than 50 citation indicates the knowledge flow among journal outlets in the current dataset (Fig. 4). The node size indicated the size of publications in a journal, while the node colour was scaled to the averaged citation per document. Edges between nodes indicated citation linkage between journals. The thicker an edge is, the more a journal cited or was cited the other journal. Public health and policy journals were clustered at the west of the network. The core journals of this cluster were the *Lancet Infectious Disease*, a medical journal that aims to influence health policies, and *PLOS One*, a non-specialized interdisciplinary journal. Medical journals were clustered at the east of the network. The west and the east clusters were connected by three journals located at the centre of the network, including *Social Science and Medicine*, *Antimicrobial Resistance and Infection Control*, and *BMJ Open*. Specifically, *Journal of Antimicrobial Chemotherapy* was clustered within the medical journals, offering knowledge and insights predominantly to medical journals in the last decades.

**Fig. 5:**
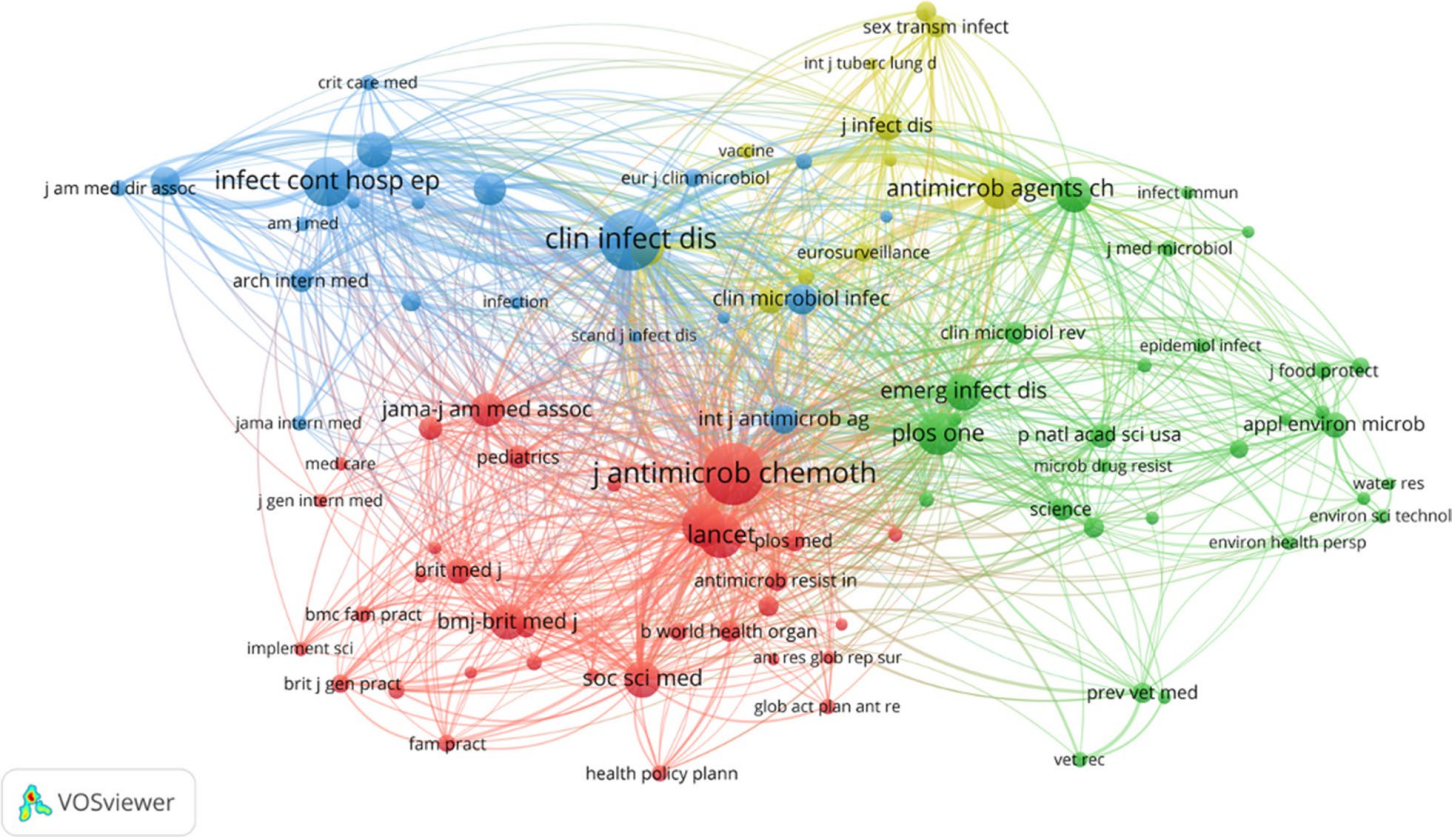
The co-citation network of journals that received more than 50 citations. The node size indicated the size of co-citation of a journal, while the node colour indicates the category of a journal. Edges between nodes indicated co-citation linkage between journals. The thicker an edge is, the more a journal was co-cited with the other journal

A co-citation network of journals was also analysed to identify clusters of journals that form the inner core of the social science research on AMR (Fig. 5). The node size indicated the size of co-citation of a journal, while the node colour indicates the category of a journal. Edges between nodes indicated co-citation linkage between journals. The thicker an edge is, the more a journal was co-cited with the other journal by research documents. As demonstrated in Fig. 5, two dimensions that categorised journals into 4 clusters emerged. The horizontal dimension indicated a spectrum from internal medicine to general medicine and environmental health science. The vertical dimension indicated a spectrum from medical sciences to social and public health sciences.

**Fig. 6:**
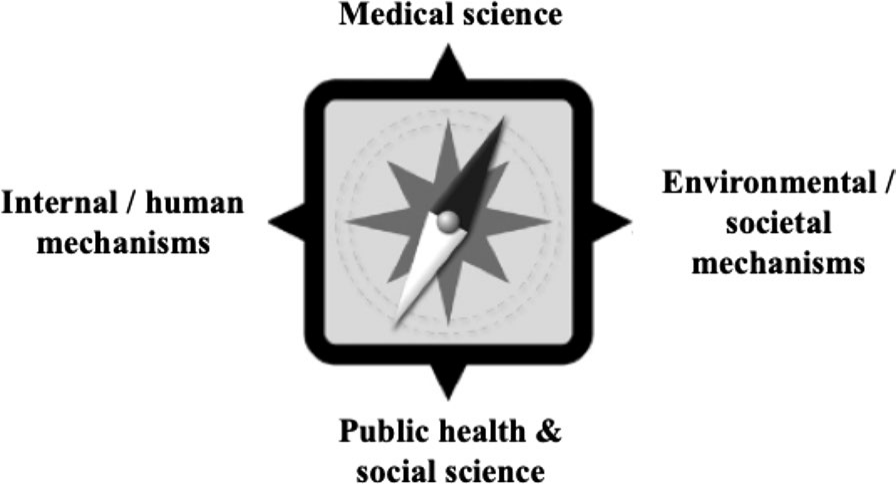
The antimicrobial resistance one-health studies compass

Remarkably, the co-citation network of journals demonstrates general and environmental health science was cited as a major research cluster (Green cluster) in the AMR social science research. This reflects the current trend of the one health approach in tackling infectious disease and AMR issues. Nevertheless, these journals are predominantly connected only to medical and microbial science (Yellow cluster and Blue cluster). Social science outlets (Red cluster), such as *Social Science and Medicine* and *Health Policy and Planning*, were rarely co-cited with environmental science outlets in the AMR research. This suggests that the one health approach has not been well incorporated into social and public health discourses, corresponding to the findings of the keyword co-occurrence analysis. Particularly, *Journal of Antimicrobial Chemotherapy* was located at the heart of the graph, suggesting that it was often co-cited with journals from all research clusters. This indicates that this journal often published interdisciplinary research in the last decade that attracted attention from multiple research areas (Fig. 6).

## Discussion

AMR is a global threat to the sustainability of human health and development. Social sciences can contribute to tackling this public health issue by providing domain knowledge that other sectors may not be able to provide to the field. By analysing bibliometric data of SSCI literature on AMR, this study demonstrated the landscape of social science research on AMR in the last decade and identified research gaps and future directions of AMR social science research.

Though social science research within the area of AMR has rapidly increased in the past few years, the field remains relatively new and requires continued and intense international collaborations. Findings from our authorship analysis revealed that only 31.2% of social science studies on AMR involves non-high-income countries that house 84.3% of the global population. This phenomenon may be due to several important reasons besides the obvious economic factors. Countries at lower income levels may be less aware of the AMR issues than those at higher income levels. For example, AMR is largely a neglected problem in South-East Asia Region, as only 6 of the 11 regional countries carried out national AMR surveillance up to 2014 [43]. Also, lack of laboratory capacity for AMR surveillance in less developed countries may limit the development of social science research on AMR. Many countries in Africa and South-East Asia reported challenges surrounding AMR surveillance and research due to lack of standard surveillance frameworks, weakness in laboratory testing of drug resistance, and lack of trained staff [43, 62]. Therefore, besides coordination at the policy levels [63], international collaborations among countries at different income levels should be encouraged by the academic society, which will be essential to help the development of AMR surveillance and follow-up research in less developed countries.

Another important finding that warrants attention is that regional collaboration networks of AMR social science research remains undeveloped. While WHO has been promoting political coordination in data sharing and dissemination of AMR research [43], our co-authorship network analysis reveals significant gaps of academic collaborations within WHO regions in the last decade. This is consistent with the 5C Framework for assessment of minimum collaboration utilized by Ardal et al. [63] indicating that collaborative actions require higher levels of joint arrangements and decisions than coordination. However, unlike the political ones, academic collaborations can often bypass political boundaries because they can take various forms and be fulfilled through personal networks, academic networks sharing the same interests, and existing institutional connections [64]. Therefore, regional collaborations should be encouraged at the academic level as it may in turn promote future political collaborations.

Importantly, our analyses of keywords, articles, and journals showed cohesive evidence that one health was marginally linked to social science AMR research, though it has been largely connected with medical and microbial sciences. Given that the one health approach has only been recognized as a fundamental approach to combat AMR in the past five years, this disconnection is understandable. Nevertheless, as agriculture and food sectors are expected to account for two-thirds of the future antibiotic use in the coming decade [9], it is imperative to understand why and under what conditions antimicrobials are used so extensively in these sectors [65]. Social issues surrounding AMR campaigns targeting food industries are also in need of being resolved by social sciences [66].

In fact, under the umbrella of one health, all social science disciplines can play important roles in reducing AMR. For example, economic models can likely offer optimal solutions to balance profits and risks regarding antibiotic use, while sociology can utilize qualitative methods to understand and resolve tensions between different AMR stakeholders from the institutional perspective. The disciplines of communication and psychology can focus on a more micro-level to study attitudinal and behavioural mechanisms underlying debates of antibiotic use in food and animal sectors.

Consistent with Frid-Nielson et al.’s [15] findings, our results provided further evidence that AMR has not been incorporated into the core discourses of social science disciplines. This is surprising as some of the disciplines has been well engaged in the field of public health. For example, the disciplines of communication, and health communication particularly, have greatly contributed to academic discourses in various public health and medical topics including obesity and infectious diseases [67–69]. WHO has called for programs to improve awareness of AMR through communication and education [43]. Given that these programs will inevitably involve communication media and techniques, health communication research can play an essential role in examining the impacts of media on AMR as well as revealing the public’s opinions and practices on antimicrobials via media (e.g., social media). Various health communication models such as the health belief model can be utilized to educate people on prudent antimicrobial use. These models can also help understand why the public inappropriately consume antibiotics for some common illness (e.g., influenza) when their effect is in fact very limited. Such research will inform better strategies for reducing antimicrobial use.

We only analysed bibliometric data from the SSCI WoS database, which may not reflect the complete set of research in the field. Nevertheless, as the SSCI database only includes journals that meet high research standards, the dataset ensures that our findings are based on articles that passed high standard peer-review processes and observe minimised bias.

## Conclusion

AMR is a major health threat that will become more prominent in the coming decades. The battle with AMR requires global efforts from all academic fields. Though social science research on AMR was rapidly growing in the past five years, the field is still young and requires intense international academic contributions and collaborations. The one health approach should be addressed as it provides an overarching framework for contributions from different scientific disciplines. This study demonstrates the current landscape of AMR social science research and calls for international academic efforts from the one health perspective.

## Data Availability

The datasets used and/or analysed during the current study are available from the corresponding author on reasonable request.

## Abbreviation

AMR: Antimicrobial resistance
